# Early postoperative complications after open versus laparoscopic radical prostatectomy: a retrospective cohort analysis using Clavien–Dindo classification

**DOI:** 10.25122/jml-2026-0003

**Published:** 2026-03

**Authors:** Alexandru-Ionuț Cherciu, Andrei-Cosmin Bumbea, Mihai-Cristian Persu, Mădălina-Maria Cherciu, Mihnea Cristian Firoiu, Radu Tiberiu Vrabie, Emilian Bolovan, Dragoș Mihail Arbunea, Darius Marian Brînzan, Andreea-Iuliana Ionescu, Ovidiu-Gabriel Bratu

**Affiliations:** 1 Department of Urology, Carol Davila University of Medicine and Pharmacy, Bucharest, Romania; 2 Department of Urology, University Emergency Hospital Bucharest, Bucharest, Romania; 3 Department of Oncological Radiotherapy and Medical Imaging, Carol Davila University of Medicine and Pharmacy, Bucharest, Romania; 4 Department of Medical Oncology, Coltea Clinical Hospital, Bucharest, Romania

**Keywords:** prostate cancer, radical prostatectomy, Clavien–Dindo, complications, laparoscopic surgery, open surgery

## Abstract

Early postoperative complications significantly influence short-term recovery after radical prostatectomy. Although laparoscopic radical prostatectomy (LRP) provides perioperative advantages compared with open radical prostatectomy (ORP), its impact on early morbidity remains uncertain. This study aimed to compare 30-day postoperative complications between ORP and LRP and to identify independent predictors of early morbidity. This retrospective two-center cohort included 149 consecutive patients undergoing ORP (*n* = 98) or LRP (*n* = 51) between 2022 and 2024. Complications were graded using the Clavien–Dindo classification. Multivariable logistic regression models evaluated independent predictors, including age, body mass index (BMI), pelvic lymph node dissection (PLND), and surgical approach. LRP was associated with shorter operative time (193.1 ± 18.9 vs 231.0 ± 27.0 minutes; *P* < 0.001), lower estimated blood loss (396.1 ± 114.4 vs 534.7 ± 108.3 mL; *P* < 0.001), and shorter hospitalization (4.92 ± 1.07 vs 6.50 ± 1.12 days; *P* < 0.001). Early complications occurred in 18.1% of patients (21.4% ORP vs 11.8% LRP; absolute difference 9.6%; 95% CI, –2.4% to 21.6%; *P* = 0.146). The distribution of complication severity was similar between groups. In multivariable analysis, BMI independently predicted early complications (OR = 1.76 per kg/m^2^; *P* < 0.001), and PLND was associated with increased risk in the extended model (OR = 5.51; *P* = 0.009). The surgical approach was not independently associated with complications. Despite clear perioperative advantages of LRP, early postoperative morbidity appeared more strongly associated with BMI and PLND than with surgical access. Larger prospective studies are warranted.

## Introduction

Radical prostatectomy (RP) remains a standard curative treatment for localized and selected locally advanced prostate cancer. Postoperative recovery and early morbidity are influenced by both patient-related factors and surgical technique. Over the past two decades, minimally invasive approaches have gained increasing acceptance due to improved visualization, reduced soft-tissue trauma, and potentially faster postoperative recovery compared with open retropubic prostatectomy [1–3]. However, whether these technical advantages translate into reduced early postoperative complications remains a matter of debate.

Early postoperative morbidity, typically defined as complications occurring within 30 days after surgery, represents a clinically relevant outcome, as it may affect hospital stay, readmission risk, recovery trajectory, and overall healthcare utilization [4]. The Clavien–Dindo classification provides a standardized, reproducible system for grading surgical complications by therapeutic consequences, enabling meaningful comparisons across studies and surgical techniques [5].

Several comparative studies have reported that laparoscopic radical prostatectomy (LRP) is associated with reduced blood loss and lower transfusion rates compared with open radical prostatectomy (ORP), while maintaining comparable overall complication rates [6–8]. At the same time, accumulating evidence suggests that patient-related factors—particularly body mass index (BMI)—may exert a stronger influence on early morbidity than the surgical approach itself [9,10]. In addition, pelvic lymph node dissection (PLND), although important for accurate oncologic staging, has been associated with increased perioperative morbidity due to the broader surgical field and the risk of lymphocele formation, bleeding, or infectious complications [11].

Given these considerations, contemporary analyses should not focus solely on surgical access but also account for patient characteristics and procedural extent when evaluating early outcomes. Therefore, the present study aimed to compare 30-day postoperative complications between ORP and LRP in a contemporary two-center institutional cohort, using the Clavien–Dindo classification, and to identify independent predictors of early postoperative morbidity through multivariable logistic regression analysis.

## Material and Methods

### Study design and population

This retrospective cohort study included 149 consecutive patients who underwent radical prostatectomy between January 2022 and December 2024 at two urological care facilities: a public tertiary-level university hospital and a private clinic.

During the study period, the two institutions functioned as parallel clinical settings. All procedures were performed by the same experienced uro-oncologic surgical team across both centers. Therefore, differences between centers reflected administrative structure and referral pathways rather than variations in surgical personnel or operative philosophy.

Open radical prostatectomy (ORP) was performed in 98 patients and laparoscopic radical prostatectomy (LRP) in 51 patients. The choice of surgical approach was determined based on the surgeon’s expertise, anatomical considerations, and patient preference following standardized preoperative counselling. No randomization was performed.

Eligibility criteria were:

- Age ≥18 years
- Histologically confirmed prostate adenocarcinoma
- Radical prostatectomy performed between January 2022 and December 2024 using either ORP or LRP
- Availability of complete perioperative data and documented 30-day follow-up for complication assessment

Exclusion criteria included:

- Salvage radical prostatectomy after prior radiotherapy or focal therapy
- Concomitant major surgical procedures unrelated to prostatectomy
- Incomplete medical records precluding accurate evaluation of perioperative outcomes

All consecutive eligible patients during the study period were included in the final analysis. The study was conducted in accordance with the STROBE recommendations for observational cohort studies.

### Data collection

Baseline demographic and clinical variables were extracted from institutional medical records. These included:

- Age
- Body mass index (BMI, kg/m^2^)
- Comorbidities (hypertension, diabetes mellitus, and coronary artery disease)
- Preoperative prostate-specific antigen (PSA)
- Prostate volume
- PSA density
- Clinical tumor stage (cTNM)
- Biopsy International Society of Urological Pathology (ISUP) grade group

Perioperative variables included:

- Operative time (minutes)
- Estimated blood loss (mL)
- Length of postoperative hospital stay (days)
- Performance of pelvic lymph node dissection (PLND)

PLND was performed according to contemporary oncologic indications and the surgeon’s discretion.

### Outcome measures

The primary endpoint was the occurrence of any postoperative complication within 30 days following surgery.

Complications were classified according to the Clavien–Dindo classification system [5], which grades adverse events based on the therapeutic intervention required:

- Grade I–II: minor complications
- Grade III–V: major complications

Secondary endpoints included:

- Operative time
- Estimated blood loss
- Length of hospital stay

All complications were identified through review of inpatient records and structured postoperative follow-up documentation.

### Statistical analysis

Continuous variables were assessed for normality using the Shapiro–Wilk test. Data were presented as mean ± standard deviation for normally distributed variables or median (interquartile range) when appropriate.

Between-group comparisons (ORP vs LRP) were performed using:

- Student’s *t*-test for normally distributed continuous variables
- Mann–Whitney U test for non-normally distributed variables
- Chi-square test or Fisher’s exact test for categorical variables

Absolute differences in complication rates between groups were calculated, and corresponding 95% confidence intervals (CI) were reported.

To identify independent predictors of early postoperative complications, multivariable logistic regression was performed in a staged manner:

- **Model A:** surgical approach, age, and BMI
- **Model B:** Model A plus pelvic lymph node dissection (PLND)

Variables were selected based on clinical relevance and to limit model complexity relative to the number of outcome events. Adjusted odds ratios (OR) with 95% confidence intervals were calculated. Statistical significance was defined as a two-sided *P* value <0.05. All statistical analyses were performed using JASP (version 0.95).

## Results

A total of 149 patients were included in the analysis, of whom 98 (65.8%) underwent open radical prostatectomy (ORP) and 51 (34.2%) underwent laparoscopic radical prostatectomy (LRP). All consecutive patients during the study period met the inclusion criteria and had a complete 30-day follow-up. No patients were excluded.

### Baseline characteristics

Baseline demographic, preoperative, and oncological characteristics were comparable between groups (Table 1).

**Table 1:** Baseline characteristics of patients undergoing ORP and LRP

Variable	ORP (n = 98)	LRP (n = 51)	Test	*P* value
**Age (years)**	63.87 ± 7.71	65.14 ± 7.16	t-test	0.330
**BMI (kg/m^2^)**	27.45 ± 1.61	26.01 ± 1.94	t-test	**<0.001**
**PSA (ng/mL)**	13.32 ± 11.02	12.90 ± 10.43	Mann–Whitney U	0.821
**Prostate volume (mL)**	57.66 ± 14.76	56.37 ± 13.45	t-test	0.603
**PSA-density**	0.223 ± 0.154	0.220 ± 0.133	Mann–Whitney U	0.904
**Hypertension (%)**	39 (39.8%)	21 (41.2%)	χ^2^	0.870
**Diabetes (%)**	28 (28.6%)	14 (27.5%)	χ^2^	0.885
**Coronary artery disease (%)**	24 (24.5%)	14 (27.5%)	χ^2^	0.694
**Clinical T stage**			χ^2^	0.854
– cT1	19 (19.4%)	9 (17.6%)		
– cT2	68 (69.4%)	38 (74.5%)		
– cT3+	11 (11.2%)	4 (7.8%)		
**ISUP grade (biopsy)**			χ^2^	0.350
– Grade 1	12 (12.2%)	13 (25.5%)		
– Grade 2	28 (28.6%)	12 (23.5%)		
– Grade 3	30 (30.6%)	12 (23.5%)		
– Grade 4–5	28 (28.6%)	14 (27.5%)		
**PLND performed (%)**	12 (12.2%)	12 (23.5%)	χ^2^	0.075

Continuous variables are presented as mean ± SD or median (IQR) depending on distribution. Categorical variables are shown as numbers (%).

**Figure 1:**
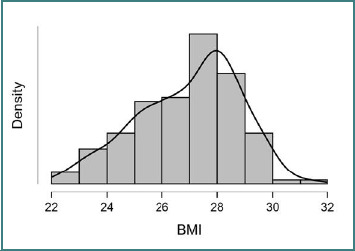
Histogram with an overlaid kernel density curve demonstrating the distribution of BMI in the study cohort. The BMI distribution approximates normality, with most patients clustering between 25 and 30 kg/m^2^. This variable was included in the multivariable models due to its significant association with early postoperative complications.

There were no statistically significant differences between the ORP and LRP groups in terms of age (63.87 ± 7.71 vs 65.14 ± 7.16 years; *P* = 0.330). BMI was significantly higher in the ORP group compared to the LRP group (27.45 ± 1.61 vs 26.01 ± 1.94 kg/m^2^; *P* < 0.001; Figure 1).

Preoperative PSA values were comparable between groups (13.32 ± 11.02 in ORP vs 12.90 ± 10.43 ng/dl in LRP; *P* = 0.821). Prostate volume (57.66 ± 14.76 vs 56.37 ± 13.45 mL; *P* = 0.603) and PSA density (0.223 ± 0.154 vs 0.220 ± 0.133; *P* = 0.904) were also similar.

The prevalence of comorbidities did not differ significantly between groups, including hypertension (39.8% vs 41.2%; *P* = 0.870), diabetes mellitus (28.6% vs 27.5%; *P* = 0.885), and coronary artery disease (24.5% vs 27.5%; *P* = 0.694).

Clinical tumor stage distribution (*P* = 0.854) and biopsy ISUP grade (*P* = 0.350) were comparable between approaches.

Pelvic lymph node dissection was performed more frequently in the LRP group (23.5%) than in the ORP group (12.2%), although this difference did not reach statistical significance (*P* = 0.075).

### Perioperative outcomes

**Figure 2:**
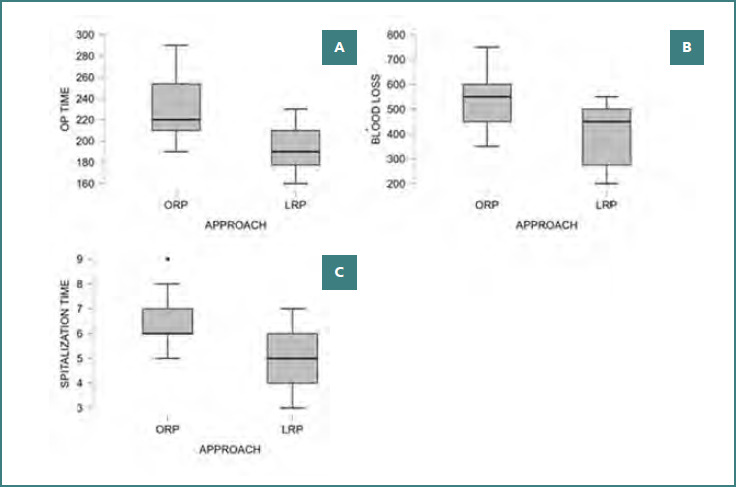
Perioperative outcomes following ORP and LRP. A, operative time (minutes); B, estimated blood loss (mL); C, postoperative hospitalization time (days) for ORP and LRP. LRP demonstrated significantly shorter operative time, reduced intraoperative blood loss, and shorter hospitalization compared with ORP (all P < 0.001). Boxes represent the IQR, the horizontal line indicates the median, whiskers show 1.5× IQR, and outliers are displayed as individual points.

Perioperative outcomes are summarized in Table 2 and Figure 2A-C. LRP was associated with significantly shorter operative time compared with ORP (193.1 ± 18.9 vs 231.0 ± 27.0 minutes; mean difference 37.9 minutes; *P* < 0.001). Estimated blood loss was significantly lower in the LRP group (396.1 ± 114.4 mL) compared with the ORP group (534.7 ± 108.3 mL; *P* < 0.001). Length of hospital stay was also significantly shorter after LRP (4.92 ± 1.07 vs 6.50 ± 1.12 days; *P* < 0.001). In linear regression analysis, surgical approach independently predicted length of hospitalization (β = –1.587; *P* < 0.001), whereas age, BMI, and PLND were not significant predictors.

**Table 2:** Perioperative outcomes according to surgical approach (ORP vs LRP)

Outcome	ORP (n = 98)	LRP (n = 51)	Test	Effect size	*P* value
**Operative time (min)**	231.0 ± 27.0	193.1 ± 18.9	t-test	d = 1.28	**<0.001**
**Estimated blood loss (mL)**	534.7 ± 108.3	396.1 ± 114.4	Mann–Whitney U	d = 1.52	**<0.001**
**Length of stay (days)**	6.50 ± 1.12	4.92 ± 1.07	Mann–Whitney U	d = 1.60	**<0.001**

### Early postoperative complications

**Figure 3:**
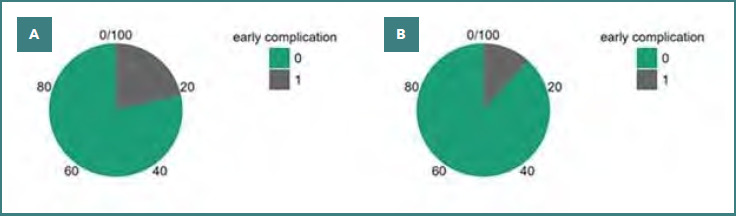
Distribution of early postoperative complications in ORP and LRP groups. Pie charts show the proportion of patients with and without early (30-day) complications following (A) open radical prostatectomy and (B) laparoscopic radical prostatectomy. Although the incidence of early complications was higher in ORP (21.4%) compared with LRP (11.8%), the difference did not reach statistical significance (**χ**^2^ test, P = 0.146).

Within 30 days after surgery, early postoperative complications occurred in 27 patients (18.1%). Complications were observed in 21 of 98 ORP patients (21.4%) and 6 of 51 LRP patients (11.8%). The absolute difference in complication rates was 9.6% (95%CI, –2.4% to 21.6%), which did not reach statistical significance (*P* = 0.146). The distribution of early complications is summarized in Figure 3AB.

**Figure 4:**
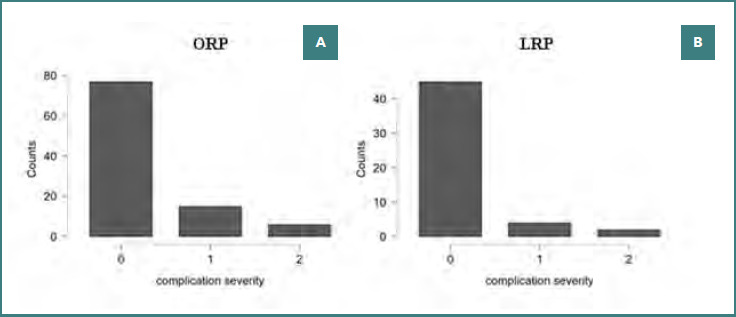
Bar plots showing the distribution of complication severity according to the Clavien–Dindo classification among patients undergoing (A) ORP and (B) LRP. Severity categories include: 0 = no complication, 1 = minor complication (Clavien I–II), and 2 = major complication (Clavien III–V). No statistically significant differences were observed between groups in either overall or severity-specific complication rates (P = 0.341).

The distribution of complication severity according to the Clavien–Dindo classification did not differ significantly between groups (χ^2^ = 2.15, df = 2; *P* = 0.341). Major complications (Clavien ≥III) were infrequent in both groups. Complication severity according to the Clavien–Dindo system was similar for ORP and LRP (Figure 4AB).

### Multivariable analysis

**Figure 5:**
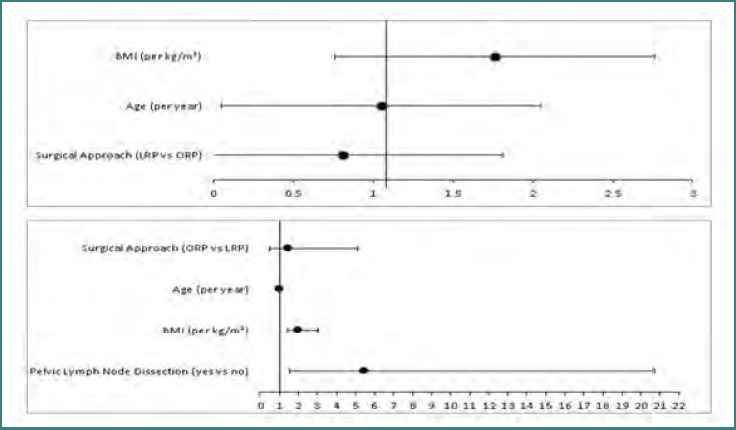
Forest plots showing adjusted ORs and 95% CIs for predictors of early postoperative complications. A, Primary model adjusting for surgical approach, BMI, and age. BMI was the only independent predictor of early complications; B, Secondary model additionally adjusting for PLND, in which both PLND and BMI remained significant predictors. The surgical approach was not significantly associated with complication risk in either model. The vertical reference line represents OR = 1.

Multivariable logistic regression identified BMI and, in the extended model, PLND as independent predictors of early complications (Figure 5AB).

### Primary logistic regression model

In multivariable logistic regression, adjusting for age and BMI (as seen in Table 3), surgical approach (LRP vs ORP) was not independently associated with early postoperative complications (adjusted OR = 0.81; 95% CI, 0.26–2.32; *P* = 0.699). BMI was independently associated with increased odds of early complications (adjusted OR = 1.76 per kg/m^2^; 95% CI, 1.29–2.53; *P* < 0.001). Age was not significantly associated with complication risk (adjusted OR = 1.05; 95% CI, 0.99–1.12; *P* = 0.129).

**Table 3:** Multivariable logistic regression models assessing predictors of early postoperative complications

A. Primary model (Approach + Age + BMI)				
**Predictor**	**Estimate**	**OR**	**95% CI**	***P* value**
**Intercept**	−20.083	–	–	<0.001
**Approach (LRP)**	−0.214	0.81	0.26–2.32	0.699
**BMI**	0.565	1.76	1.29–2.53	<0.001
**Age**	0.047	1.05	0.99–1.12	0.129
**B. Secondary model (Approach + Age + BMI + PLND)**				
**Predictor**	**Estimate**	**OR**	**95% CI**	***P* value**
**Intercept**	−23.646	–	–	<0.001
**Approach (ORP)**	0.410	1.51	0.50–5.13	0.485
**PLND**	1.706	5.51	1.54–20.72	0.009
**BMI**	0.691	1.99	1.42–2.96	<0.001
**Age**	0.038	1.04	0.98–1.11	0.243

Model A adjusts for age and BMI. Model B includes PLND as a clinically relevant covariate. OR with 95% CI are provided. The reference category for the surgical approach was ORP between models.

### Secondary model including PLND

In a secondary model that additionally adjusted for PLND (summarized in Table 3), pelvic lymph node dissection was independently associated with increased odds of early complications (adjusted OR = 5.51; 95% CI, 1.54–20.72; *P* = 0.009). BMI remained significantly associated with complication risk (adjusted OR = 1.99 per kg/m^2^; 95% CI 1.42–2.96; *P* < 0.001). Surgical approach remained non-significant (adjusted OR = 1.51; 95% CI, 0.50–5.13; *P* = 0.485).

### Supplementary model including comorbidities

An exploratory model including surgical approach and cardiovascular comorbidities (hypertension, diabetes mellitus, and coronary artery disease) did not demonstrate statistically significant independent associations (all *P* > 0.05). To limit model complexity relative to the number of outcome events, BMI was not included in this model.

Given the limited number of events (*n* = 27), regression estimates should be interpreted cautiously.

## Discussion

This retrospective two-center cohort study evaluated early (30-day) postoperative complications following open and laparoscopic radical prostatectomy in a contemporary institutional setting. Consistent with prior literature, LRP was associated with significantly shorter operative time, reduced intraoperative blood loss, and shorter hospitalization [6,12,13]. However, although the crude complication rate was numerically lower after LRP, the surgical approach was not identified as an independent predictor of early postoperative complications in multivariable analysis.

The absolute difference in complication rates between the approaches was 9.6%, with a 95% confidence interval of –2.4% to 21.6%. This interval includes both a small potential disadvantage and a clinically meaningful benefit, indicating that moderate differences cannot be definitively excluded. Therefore, the absence of statistical significance should not be interpreted as evidence of equivalence, particularly given the limited number of outcome events. Notably, BMI differed significantly between groups, with higher values observed in the ORP cohort. Given the independent association between BMI and early complications, this imbalance may partially account for the higher crude complication rate observed in the ORP group and underscores the importance of multivariable adjustment when comparing surgical approaches in observational settings.

Our findings suggest that patient-related and procedural factors may exert a greater influence on early morbidity than surgical access alone. BMI consistently emerged as an independent predictor of early complications across multivariable models. Obesity has been associated with increased operative complexity, prolonged tissue dissection, impaired wound healing, and systemic inflammatory alterations that may predispose patients to postoperative adverse events [9,14,15]. The magnitude of association observed in this cohort is in line with previous reports demonstrating increased perioperative risk among overweight and obese patients undergoing radical prostatectomy.

Pelvic lymph node dissection was independently associated with a higher risk of early complications in the extended model. PLND increases operative extent and may predispose patients to lymphocele formation, bleeding, or infectious complications [11,16]. Nevertheless, the wide confidence interval around the PLND estimate reflects limited event numbers and indicates that the strength of this association should be interpreted cautiously.

Importantly, the surgical approach remained non-significant after adjustment for BMI and PLND. These findings are consistent with comparative studies suggesting that early postoperative morbidity may depend more on patient characteristics and procedural complexity than on access technique alone [8,17]. While minimally invasive surgery provides clear perioperative advantages in terms of blood loss and hospital stay, its impact on short-term complication rates may be attenuated when procedures are performed by experienced surgeons within standardized institutional pathways.

The overall complication rate of 18.1% and the low incidence of major (Clavien ≥ III) events observed in this cohort are consistent with contemporary series using the standardized Clavien–Dindo classification [7,10]. This supports the safety of both ORP and LRP in high-volume settings when performed by experienced surgical teams.

### Limitations

Several limitations should be acknowledged. First, the retrospective, non-randomized design introduces the possibility of residual confounding. Although multivariable adjustment was performed, unmeasured factors influencing surgical selection or patient risk may persist. Second, the relatively small number of complication events (*n* = 27) limits statistical power and may reduce the stability of regression estimates. Consequently, clinically relevant differences between approaches cannot be definitively excluded.

Third, although all procedures were performed by the same experienced surgical team across both institutions, center-level differences related to infrastructure or perioperative care pathways cannot be entirely ruled out. Finally, only early (30-day) complications were evaluated; long-term functional and oncologic outcomes were beyond the scope of this analysis.

## Conclusion

In this retrospective two-center cohort, laparoscopic radical prostatectomy demonstrated clear perioperative advantages, including shorter operative time, reduced blood loss, and shorter hospitalization. Although the incidence of early (30-day) postoperative complications was numerically lower following LRP, the surgical approach was not independently associated with early morbidity after multivariable adjustment.

Body mass index and pelvic lymph node dissection were independently associated with increased odds of early complications, underscoring the importance of patient-related factors and procedural extent in perioperative risk assessment. These findings suggest that early postoperative outcomes may be influenced more by individual risk profile and surgical complexity than by access technique alone.

Given the limited number of complication events, clinically meaningful differences between surgical approaches cannot be definitively excluded. Larger prospective studies are warranted to further clarify the relative contribution of surgical approach and patient characteristics to early postoperative morbidity.

## Data Availability

Further data is available from the corresponding author upon reasonable request.
